# Delayed sequential lung, acral cutaneous and brain metastases after radical nephrectomy for clear cell renal cell carcinoma: a case report

**DOI:** 10.3389/fonc.2026.1817630

**Published:** 2026-10-19

**Authors:** Péter Álmos Rajhóczki, Viktor Kardos, Rézan Watti, Marcell Juhász, Magdolna Horváth, Dávid Pócs, Jezdancher Watti

**Affiliations:** 1 Bugát Pál Kórház, Gyöngyös, Hungary; 2 Mátrai Gyógyintézet, Gyöngyös, Hungary; 3 Zala Megyei Szent Rafael Korhaz, Zalaegerszeg, Hungary; 4 Szegedi Tudomanyegyetem, Szeged, Hungary

**Keywords:** brain metastasis, clear cell renal cell carcinoma, cutaneous metastasis, lung metastasis, metastatic RCC (mRCC), renal cell carcinoma

## Abstract

Renal cell carcinoma may recur after nephrextomy. Approximately 25-30% of all RCC patients develop metastases, most commonly involving the lungs, bones and brain. Cutaneous metastases are exceptionally rare (about 3% of all RCC patiens) and are associated with a favorable prognosis. While the surgical interventions remain the cornerstone of the management of RCCs, chemotherapy and biological therapies are also available, therefore a complex approach is a must in order to reach the best possible outcome. This is a case report of a 62-year-old male patient with obesity, type 2 diabetes mellitus and smoking history, who underwent a laparoscopic transperitoneal radical right nephrectomy in 2019. Later the histological examination confirmed clear cell renal cell carcinoma. During the course of the disease, he developed multiple metastases involving the lungs, the skin and the brain. He received oncological therapies as well as surgeries – involving robot-assisted thoracoscopic surgery and CyberKnife surgery - and tolerated the therapeutic methods well. This case emphasizes that even small acral lesions in patients with prior renal cell carcinoma may represent metastatic disease and demonstrates the value of prolonged surveillance and multidisciplinary treatment in rare delayed metastatic presentations.

## Background

The malignant tumors of the kidneys have an annual incidence of 430,000 cases and a mortality of 180,000 (1). The most common tumor of the kidney parenchyma is renal cell carcinoma (RCC). RCC can be divided into more than 10 distinct histopathological subtypes and the exact histological diagnosis has a major impact on survival (2–5). More than 50% of RCC cases are detected incidentally during thoracoabdominal imaging performed for other indications (6, 7). The classic triad of hematuria, loin pain, and abdominal mass is rare and predicts a poor prognosis (8). 20% of patients present primarily with paraneoplastic syndromes, usually indicating synchronous metastases at the time of the diagnosis (9). The Fuhrman grading system remains frequently used, nevertheless recent studies suggest that it requires revision and incorporation of molecular biological behavior (10, 11).

Distinct histopathological subtypes of RCC arise from heterogeneous molecular pathomechanisms (12, 13). Novel therapies act on these misregulated pathways (14, 15). In the present case ipilimumab, nivolumab and sunitinib were used.

Metastases develop in 25–30% of all cases during the disease course, most commonly involving the lungs, bones, and brain, respectively: 43.6%, 20-35% and 10-15% (16–18). RCC primarily spreads through the bloodstream, although lymphatic dissemination has also been reported. Radical surgery mitigates the risk of developing a metastasis, yet nearly 40% of patients with localized RCC who undergo resection, subsequently develop distant metastases (19). The management of brain metastases has advanced considerably in recent years, increasingly incorporating immunochemotherapy combined with radiotherapy (20). Cutaneous metastases from RCC are exceedingly rare, occurring in about only 3% of cases, and are generally associated with a favorable prognosis. Acral metastases are even more infrequent, and the available literature is limited (21). This makes our case report novel and clinically relevant. The novelty of this case lies in the delayed, sequential appearance of metastases, with prolonged survival achieved through repeated multidisciplinary interventions. We hope to provide insight into the successful treatment of an unusual, metastatic renal cell carcinoma.

Despite substantial advances in diagnostics and treatment modalities in recent years, RCC continues to pose formidable challenges for clinicians (22).

## Case presentation

The patient of this study was a 62-year-old man, born in 1964, with a medical history notable for obesity (BMI of 41) and type 2 diabetes mellitus. He reported occasional alcohol consumption and a remote smoking history equivalent to 50 pack-years (23). In **Table 1** the key milestones in the patient’s medical history are summarized. Notably, he presented to the Urological Department in August 2019 (at the age of 55) for hematuria. Abdominal-pelvic ultrasonography at that time revealed a 7-cm large, echodense, inhomogeneous lesion inseparable from the parenchyma in the upper pole of the right kidney.

**Table 1:** The chronological sequence of events in the course of the treatment.

Time	Event
2019 August	Diagnosis of renal cell carcinoma
4 months after diagnosis	Laparoscopic transperitoneal radical right nephrectomy
50 months after diagnosis	Computed tomography (CT) scan revealing a metastatic lesion in the lower right lobe of the lungs
51 months after diagnosis	PET-CT confirming the metastatic lesion of the lung
53 months after diagnosis	RATS lobectomy for resection of the right lower lobe metastasis
54 months after diagnosis	Amputation of the distal phalanx of the right I. finger – later histologically proven to be a cutaneous metastasis
56–58 months after diagnosis	4 cycles of ipilimumab plus nivolumab immunochemotherapy
59 months after diagnosis	Brain magnetic resonance imaging (MRI)
60 months after diagnosis	Thoracoabdominal CT excluded progression
62–65 months after diagnosis	Nivolumab monotherapy
63 months after diagnosis	Brain MRI because of worsening neurological status
65 months after diagnosis	Stereotactic brain radiotherapy (3 x 9 Gy)
74 months after diagnosis until today	Cabozantinib monotherapy

Subsequent thoracoabdominal computed tomography (CT) confirmed a mass measuring 63 × 80 × 70 mm in the right renal pole. The CT scan, along with a bone scan was also required for the proper pre-operative staging and to rule out possible metastases at that time.

In December 2019, the patient underwent a laparoscopic transperitoneal radical right nephrectomy. Later, the histopathological examination confirmed clear cell renal cell carcinoma (ccRCC) of the previously mentioned size. The tumor was graded as Fuhrman G3, pT3aNxM0. Microscopy showed alveolar arrangements of clear cells with delicate vasculature. Surgical margins were free of tumor. Immunohistochemical analysis of the primary tumor is not available.

Thereafter, the patient showed no symptoms and required no further oncological therapy, but remained under regular urological observation without evidence of metastases. According to the oncoteam decision, these follow-ups consisted of physical examinations, abdominal sonography and thoracoabdominal CT scans every 6 months. During these CT scans, in October 2023 -at the age of 59- a large (47 × 53 mm) lesion in the right lung was detected, simultaneously with smaller bilateral pulmonary metastases, furthermore mediastinal and right hilar lymphadenopathy, and a focal right adrenal gland lesion of indeterminate nature, raising suspicion for metastasis (**Figure 1**). Four weeks later, a CT-guided core biopsy histologically confirmed metastatic renal cell carcinoma in the lungs (**Figure 2**). A complementary 18F-FDG PET-CT in November 2023 demonstrated FDG avid malignancy in the lungs without any pathological abnormalities elsewhere, confirming the lesion in the right adrenal gland benign.

**Figure 1:**
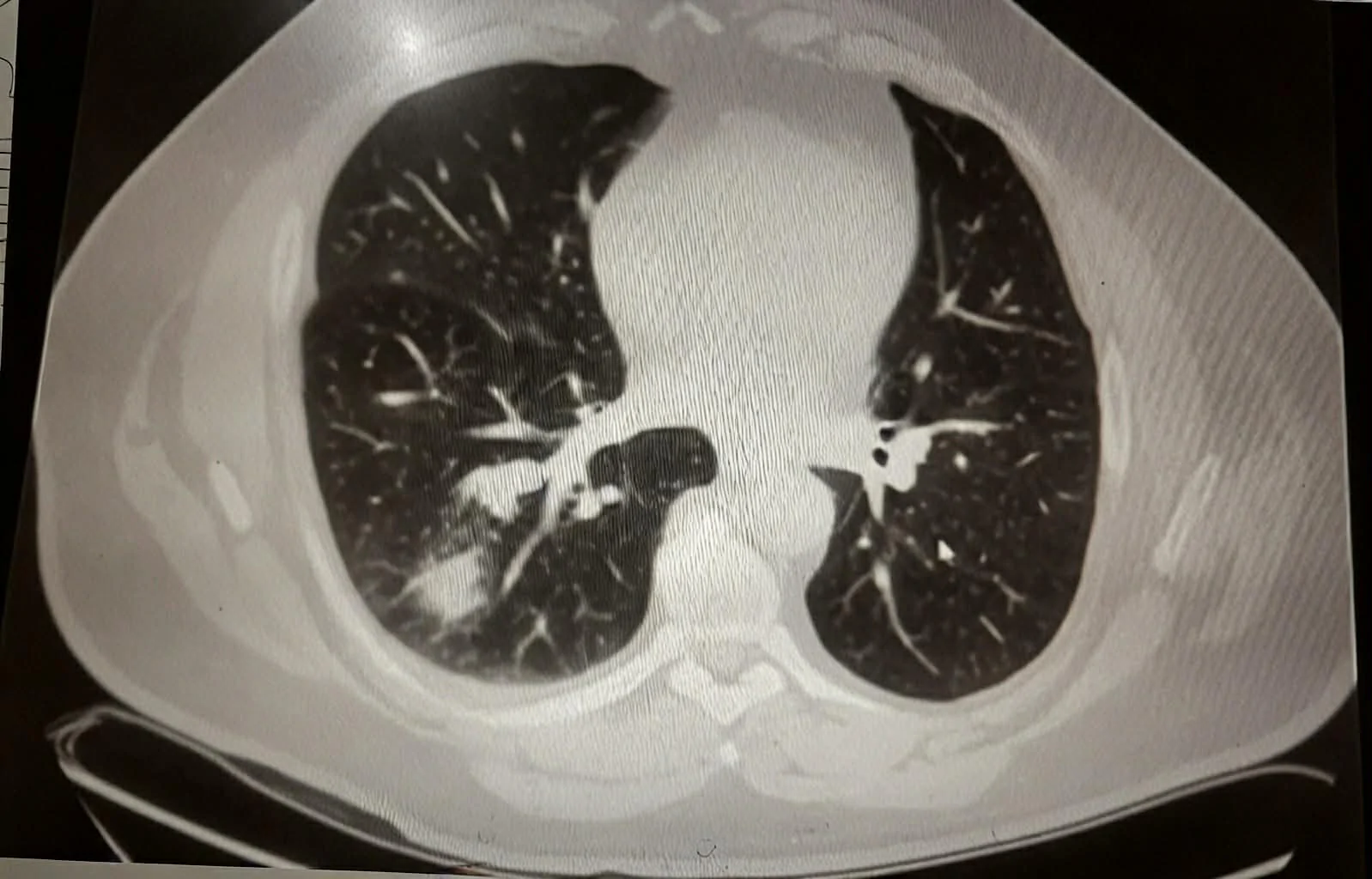
Contrast-enhanced thoracic CT scan indicating a 47 x 53 mm metastatic lesion in the right lower lobe of the lung, detected during routine surveillance imaging in October 2023.

**Figure 2:**
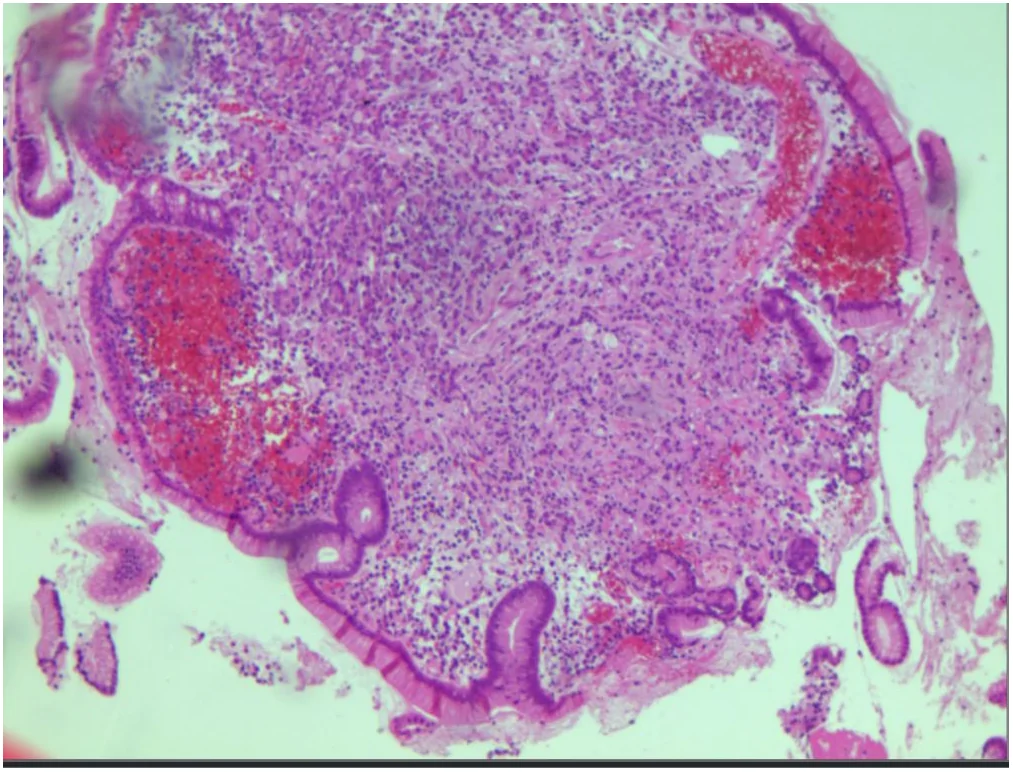
Histopathological appearance of the resected pulmonary lesions consistent with metastatic clear cell renal cell carcinoma (hematoxyllin-eosin stain), showing nests of clear cells.

An oncological-thoracic surgical consultation recommended robotic-assisted thoracoscopic surgery (RATS) for the hypermetabolic, likely malignant lesion in the right lower lobe identified on PET-CT, deeming additional distant metastases improbable (24).

The surgical procedure was performed on January 20, 2024, RATS lobectomy for resection of the right lower lobe metastasis was done. The histopathological evaluation of the pulmonary lesion demonstrated metastatic clear cell renal cell carcinoma with morphology concordant with the primary renal tumor. Immunohistochemically, the tumor cells were PAX8, CD10 and CAIX positive, CK7 and CK20 negative. Invasion of the blood vessels was also observed. Surgical margins and all sampled lymph nodes were free of tumor.

The patient was subsequently transferred to his prior pulmonology department, where he reported pain and a pea-sized granulomatous wound on the distal phalanx of the right first finger - sustained days earlier in his home while doing daily chores (**Figure 3**). On examination, the lesion appeared as a rapidly enlarging, erythematous, exophytic nodule measuring approximately 20x15x15 mm. It bled easily on contact. Post-surgical recovery was uneventful from thoracic surgical and pulmonological standpoints, meaning he did not experience any loss of respiratory function; however, a hand surgical consultation was advised for the finger lesion. After his recovery from the RATS lobectomy, he was transferred to a hand surgeon. The lesion on the finger clinically appeared to be a cutaneous cancer. An X-ray was indicated, showing no involvement of the bones. Because the lesion was clinically suspicious for malignancy, located on the distal phalanx with limited soft-tissue reserve, and considered unsuitable for simple excision with reliable margin control and wound closure, amputation at the base of the distal phalanx was selected after discussion of treatment options with the patient. In February 2024, due to clinical suspicion of cutaneous tumor involvement, the distal phalanx of the finger was amputated at the base. The histopathological examination confirmed metastatic renal cell carcinoma in the specimen. Immunohistochemistry showed positivity for PAX8, Vimentin, CD10 and EMA, and negativity for CK7. The bone was not involved. Surgical margins were free of tumor.

**Figure 3:**
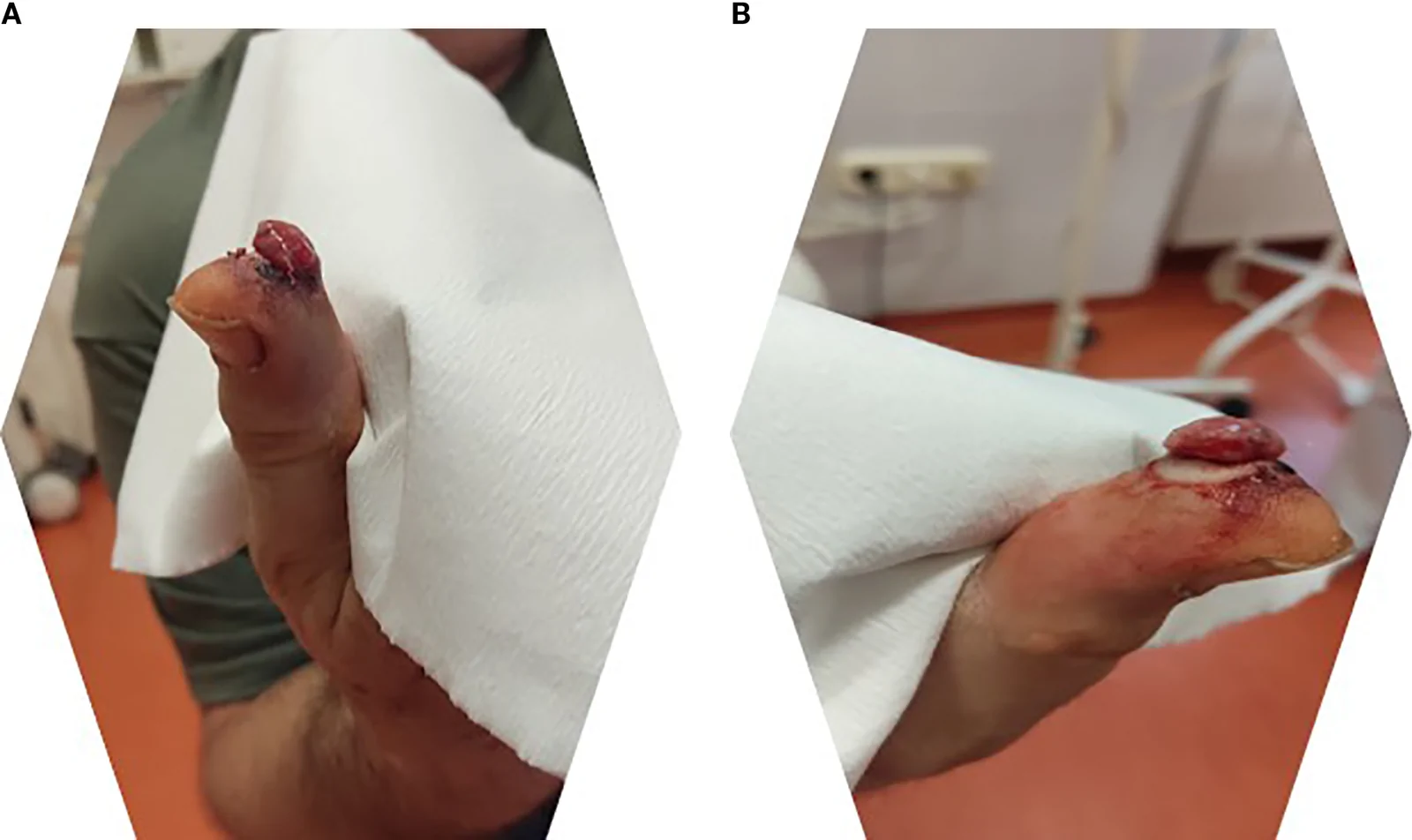
Clinical appearance of the acral lesion on the distal phalanx of the right first finger before surgery, presenting as a friable erythematous exophytic lesion.

The case was presented to the Oncological Department and ipilimumab plus nivolumab (IPI+NIVO) immunotherapy was initiated in spring 2024. During the second cycle of the IPI+NIVO therapy, *de novo* atrial fibrillation emerged. Initially an electrical cardioversion was planned, though sinus rhythm spontaneously restored (25).

After the fourth cycle of IPI+NIVO, the patient reported hallucinations, leading to a brain magnetic resonance imaging (MRI) on July 9, 2024, which revealed small focal lesions suspicious for dissemination in the left superior and medial frontal gyrus and the right inferior frontal gyrus.

A neuro-oncological consultation took place but active intervention was not advised at this point. Later transient hypoxia and altered mental status occurred and the patient was transferred to the intensive care unit for a couple of days, followed by 12 days of hospitalization, during which time he received hydration, oxygen therapy and tiapride was initiated, resulting in near-complete symptom reduction.

As of August 2024, thoracoabdominal CT showed no evidence of progression. The patient’s general condition improved, nivolumab monotherapy was resumed in October 2024. Formal RECIST-based response assessment was not available in this case; therefore the treatment response is described according to radiology reports and multidisciplinary team documentation.

In November 2024 brain MRI was repeated because of worsening neurological symptoms, which verified progression of the previously described cerebral lesions. The Neuro-Oncology and Radiosurgery Committee deemed the patient suitable for CyberKnife (26).

Between January 8–16, 2025, stereotactic brain radiotherapy (3 × 9 Gy via linear accelerator) was done, targeting four cerebral lesions. The patient tolerated the irradiation well, transitioning thereafter to oral sunitinib for nearly one year.

Series of thoracoabdominal CT, cranial CT, and MRI throughout 2025 initially showed no progression; however, minimal progression was suspected in October 2025, prompting switch to third-line cabozantinib, a VEGFR2 inhibitor, which has been well-tolerated to this date. At the last follow-up in October 2025, the patient was alive and receiving medication with good tolerance. The patient remained ambulatory and capable of self-care.

## Discussion

The principal clinical value of this case lies in the combination of delayed metachronous relapse, exceptionally rare acral cutaneous metastasis and durable disease control with outstanding survival time, achieved through sequential surgery, immunotherapy, radiotherapy and targeted therapy. An analysis of 7,386 patients with metastatic RCC found that the timing of the metastases may impact the survival time. Metachronous metastases are associated with a more favorable outcome (27).

The lung is the predominant site for metastases in RCC and in most cases is the first site for metastases. Robotic-assisted thoracic surgery (RATS) is often considered the gold standard operating method for primary lung cancer or pulmonary metastases (28). Brain metastases are also relatively common in RCC, the average survival time is less than 1 year in most cases (18). The pattern of metastatic spread in our case is typical for RCC; what makes it outstanding is the cutaneous metastasis and the relatively long survival time.

Cutaneous metastases of RCC are exceedingly rare with a reported incidence of 1.3-3.4%, which makes this case all the more intriguing (29, 30). Skin metastases usually have a favorable prognosis, although the available literature is limited (21). In an article on 28 RCC patients with cutaneous metastases, the researchers found that the median time of development of this metastasis is 5.4 years after the primary diagnosis - which is consistent with our findings (31). A review of the available English-language literature between 1990 and 2000 consisting of 81,618 cases found only 436 cutaneous metastases from urological organs (32). The published evidence on acral metastases of RCCs is insufficient, metastases involving the hand are only mentioned in less than 0.1% of all documented cases, making our report a much-needed contribution to this topic (30).

This case is distinguished not merely by the presence of metastatic RCC, but by the uncommon pattern of spread. The patient developed metachronous lung metastasis nearly four years after radical nephrectomy, followed shortly by cutaneous metastasis and brain metastasis, while maintaining meaningful disease control under sequential local and systemic treatment. The educational value of this report lies in demonstrating that delayed acral lesions in a patient with RCC must not be dismissed as benign findings.

Among patients with cancer, even the most inconspicuous lesions warrant rigorous evaluation, as they may herald serious underlying pathology. Any seemingly trivial wound or anomaly may signify reactivation or metastases of the primary malignancy, therefore clinicians must always approach these abnormalities with uppermost caution.

## Conclusion

It should be kept in mind that in RCC patients late metastases are relatively common (18).

As evidenced in this report, thorough diagnostic plans enable reliable detection, and meticulous management can substantially prolong survival time while meaningfully enhancing the quality of life.

This case highlights that clear cell renal cell carcinoma may relapse years after radical nephrectomy with an unusual sequential metastatic pattern involving the lung, acral skin and brain. Its main clinical implication is that new acral lesions in patients with a history of ccRCC, even when seemingly traumatic or benign, warrant prompt diagnostic evaluation. The favorable long-term disease control observed in this patient also illustrates the value of individualized multidisciplinary management combining metastasis-directed surgery, radiotherapy and sequential systemic treatment, underscoring the importance of these patients being treated in health centers with sufficient experience in similar cases.

## Data Availability

The original contributions presented in the study are included in the article/supplementary material. Further inquiries can be directed to the corresponding author.
